# Editorial improvement in *Epidemiologia e Serviços de Saúde* in 2024

**DOI:** 10.1590/S2237-96222024v33e20241002.en

**Published:** 2024-12-13

**Authors:** Taís Freire Galvão, Everton Nunes da Silva, Wildo Navegantes de Araújo, Jorge Otávio Maia Barreto

**Affiliations:** 1 Universidade Estadual de Campinas, Faculdade de Ciências Farmacêuticas, Campinas, SP, Brasil; 2 Ministério da Saúde, Secretaria de Vigilância em Saúde e Ambiente, Coordenação-Geral de Editoração Técnico-Científica em Vigilância em Saúde, Brasília, DF, Brasil; 3 Universidade de Brasília, Faculdade de Ciências e Tecnologias em Saúde, Brasília, DF, Brasil; 4 Fundação Oswaldo Cruz, Gerência Regional de Brasília, Brasília, DF, Brasil

The *Epidemiologia e Serviços de Saúde: revista do SUS* (RESS) underwent significant editorial changes in 2024. The editorial team was restructured, with the addition of new chief, scientific, and executive editores. The group of associate editors was also expanded with new researchers, including a peer review administrator who centralizes the management of invitations for the manuscript screened by the chief and scientific editors ^1^. By concentrating the task of identifying reviewers and sending invitations, associate editors can dedicate more attention and time to the critical evaluation of manuscripts and reviews, providing an opportunity to improve the overall quality of the peer review process. If necessary, associate editors may include other reviewers, either by searching or registering reviewers in the system.

The editorial scope of RESS was expanded to better meet the needs for evidence in health policies and systems, now accepting scientific articles in the field of public health, including epidemiology, social and human sciences in health, management, and planning, and addressing topics relevant to the SUS.

The guidelines for manuscript preparation were also revised and went through public consultation announced on RESS’s social media, which were reactivated and are available on Instagram, X/Twitter, Facebook, LinkedIn, TikTok, YouTube, all accessible via @revistadosus.

The new instructions for authores are in line with SciELO criteria ^1^, highlighting the inclusion of a declaration on the use of generative artificial intelligence, availability of article data, as well as the identification of reviewers who wish to be identified, all editors involved in manuscript processing, and the publication of reviews of approved articles. These aspects align with the best practices of open science communication and place RESS at the forefront in implementing these practices in public health.

A new submission system was adopted, housed on the ScholarOne platform (https://mc04.manuscriptcentral.com/ress-scielo), aiming to enhance editorial management resources and improve the experience for authors, reviewers, and editors. To ensure a quick and consistent transition between editorial management systems, intensive training for editors and reviewers was organized in the ‘1st RESSatona: RESS Peer Review Marathon’ in June 2024, in Brasília ^2^. This training was replicated on two other occasions - in São Paulo and Campo Grande, with the collaboration of associate editors from local institutions - and resulted in the training in the peer review process of almost 100 participants in total.

Understanding that further adjustments will need to be incorporated based on the experiences of authors, reviewers, and editors, the changes made so far have allowed for greater fluidity and consistency in the editorial process. The approval of manuscripts, previously delegated to a member of the Editorial Committee, has become the responsibility of the chief and scientific editors, bringing greater accountability to this process, given the adoption of the identification of these actors in the approved article. This modification was approved in an editorial committee meeting, during which the names appointed to editorial functions were also confirmed.

In 2024, in addition to manuscripts published in the regular volume, two special issues were published, in alignment with the objectives and guidelines of the Unified Health System (*Sistema Único de Saúde*, SUS): one on the National Vaccination Coverage Survey, providing a snapshot of the national situation regarding vaccines included in the vaccination schedule ^3^, and another on 20 years of trans visibility in Brazil, promoting the dissemination of evidence on a historically neglected population in the country ^4^. A call for papers on the 10 years of the Dietary Guidelines for the Brazilian Population was also promoted to highlight a Brazilian technology developed within the SUS ^5^. Efforts focused on quality and process improvement were aligned with the annual publication volume ^1^, reaffirming the editors’ focus on achieving results and indicators that position RESS as a prominent journal in the field of public health, but especially as a vehicle for the dissemination of evidence with different designs that can support public deliberation processes and decision-making on priority topics for governments, academia, and civil society, also contributing to the consolidation of SUS and health as a social right.

RESS reaffirms its role as the SUS journal with the strengthening of editorial practices and the dissemination of evidence for SUS. Submissions of experience reports, as well as qualitative research, are welcome, both accepted as Original articles or Research notes, providing an opportunity to disseminate necessary evidence for SUS.

## References

[1] SciELO (2024). Critérios, política e procedimentos para a admissão e a permanência de periódicos na Coleção SciELO Brasil.

[2] Silva MT, Galvao TF (2024). Systematization of peer review in Epidemiologia e Serviços de Saúde. Epidemiol Serv Saúde.

[3] MdC Tauil, Macedo LR, Maranhão AGK (2024). National Vaccination Coverage Survey and its importance amid the challenges. Epidemiol Serv Saúde.

[4] Simpson K, Benevides B (2024). 20 Years of Trans Visibility, from Mourning to Fighting!. Epidemiol Serv Saúde.

[5] Brasil. Ministério da Saúde. Secretaria de Atenção à Saúde. Departamento de Atenção Básica (2014). Guia alimentar para a população brasileira.

