# Supplementary material for: Editorial improvement in Epidemiologia e Serviços de Saúde in 2024
Source: Epidemiol Serv Saude. 2024 Dec 13;33:e20241002. doi: 10.1590/S2237-96222024v33e20241002.en (PMC11654712; doi:10.1590/S2237-96222024v33e20241002.en)
Supplement: Translation

# Translation: Aprimoramento editorial na Epidemiologia e Serviços de Saúde em 2024

**DOI:** 10.1590/S2237-96222024v33e20241002.pt

**Authors:** Taís Freire Galvão, Everton Nunes da Silva, Wildo Navegantes de Araújo, Jorge Otávio Maia Barreto

**Affiliations:** 1 Universidade Estadual de Campinas, Faculdade de Ciências Farmacêuticas, Campinas, SP, Brasil; 2 Ministério da Saúde, Secretaria de Vigilância em Saúde e Ambiente, Coordenação-Geral de Editoração Técnico-Científica em Vigilância em Saúde, Brasília, DF, Brasil; 3 Universidade de Brasília, Faculdade de Ciências e Tecnologias em Saúde, Brasília, DF, Brasil; 4 Fundação Oswaldo Cruz, Gerência Regional de Brasília, Brasília, DF, Brasil

A Epidemiologia e Serviços de Saúde: revista do SUS (RESS) passou por importante mudanças editoriais em 2024. O corpo de editores foi reformulado, com a entrada de novos editores chefe, científicos e executiva. O grupo de editores associados também recebeu incrementos e novos pesquisadores foram incorporados, incluindo uma gestora de pareceristas, que centraliza a gestão dos convites para a revisão dos manuscritos triados pelos editores chefe e científicos ^1^. Ao concentrar a tarefa de identificar pareceristas e emitir os convites, os editores associados podem dedicar mais atenção e tempo para a avaliação crítica dos manuscritos e pareceres, proporcionando oportunidade para aprimoramento da qualidade geral do processo de revisão por pares. Havendo necessidade, é facultado aos editores associados incluírem outros pareceristas, por buscas ou cadastro de revisores no sistema. 

O escopo editorial da RESS foi ampliado para atender melhor às necessidades de evidências para políticas e sistemas de saúde, passando a acolher artigos científicos no campo da saúde coletiva, incluindo epidemiologia, ciências sociais e humanas na saúde, gestão e planejamento, e que abordem temáticas relevantes para o SUS.

As instruções para a elaboração de manuscritos também foram reformuladas e passaram por consulta pública divulgada nas redes sociais da RESS, que foram reativadas e estão disponíveis nas plataformas Instagram, X/ Twitter, Facebook, LinkedIn, TikTok, YouTube, todas acessíveis por meio da @revistadosus.

As novas instruções estão em linha com os critérios SciELO ^1^, com destaque para inclusão de declaração sobre uso de inteligência artificial generativa, disponibilidade dos dados do artigo, além da identificação dos pareceristas que assim desejarem, todos os editores envolvidos no processamento do manuscrito e a publicação dos pareceres de artigos aprovados. Tais aspectos estão alinhados com as melhores práticas de comunicação da ciência aberta e colocam a RESS na vanguarda na operacionalização dessas práticas na área de saúde coletiva.

Novo sistema de submissão foi adotado, albergado na plataforma ScholarOne (https://mc04.manuscriptcentral.com/ress-scielo), a fim de aprimorar os recursos para a gestão editorial e melhorar a experiência de autores, revisores e editores. Para que a transição entre os sistemas de gestão editorial ocorresse se forma rápida e consistente, foi idealizado um treinamento intensivo para editores e revisores na ‘1ª RESSatona: maratona de revisão por pares da RESS’ em junho de 2024, em Brasília ^2^. Este treinamento foi replicado em outras duas ocasiões - em São Paulo e Campo Grande, com a colaboração de editoras associadas de instituições locais - e que resultaram no treinamento no processo de revisão por pares de quase 100 participantes, no total.

Com a clareza que outros ajustes ainda precisarão ser incorporados, a partir da experiência dos autores, revisores e editores, as mudanças realizadas até agora têm permitido maior fluidez e consistência ao processo editorial. A aprovação dos manuscritos, anteriormente delegada a um membro do Comitê Editorial, passou a ser premissa dos editores geral e científicos, trazendo maior responsabilização sobre este processo, dada a adoção da identificação desses atores no artigo aprovado. Tal modificação foi aprovada em reunião do comitê editorial, ocasião na qual os nomes apontados para as funções editoriais também foram confirmados.

Em 2024, além dos manuscritos publicados no seu volume regular, dois números especiais foram publicados, em alinhamento com os objetivos e diretrizes do Sistema Único de Saúde (SUS): um sobre o Inquérito Nacional de Cobertura Vacinal, que traz uma radiografia a situação nacional acerca dos imunizantes previstos no calendário vacinal ^3^ e outro sobre os 20 anos de visibilidade trans no Brasil, fomentando a veiculação de evidências sobre população historicamente negligenciada no país ^4^. Uma chamada sobre os 10 anos do Guia Alimentar para a População Brasileira também foi promovida para destacar uma tecnologia brasileira, desenvolvida no âmbito do SUS ^5^. Os esforços centrados na qualidade e aprimoramento do processo foram alinhados ao quantitativo de publicação anual ^1^, reafirmando o foco dos editores em prezar por resultados e indicadores que posicionem a RESS como periódico de destaque no campo da saúde coletiva, mas especialmente como veículo da disseminação de evidências, com diferentes delineamentos, que possam apoiar processos de deliberação pública e tomada de decisão em temas prioritários para governos, academia e sociedade civil, contribuindo também para a consolidação do SUS e da saúde como um direito social.

A RESS reafirma seu papel como revista do SUS com o fortalecimento das práticas editoriais e disseminação de evidências para o SUS. São bem-vindas submissões de relatos de experiências, bem como pesquisas qualitativas, ambos acolhidos na modalidade Artigo original ou Nota de pesquisa, oportunizando a veiculação de evidências necessárias ao SUS.
